# The surveillance of viral infections by the unconventional Type I NKT cell

**DOI:** 10.3389/fimmu.2024.1472854

**Published:** 2024-09-17

**Authors:** Varshini Rajashekar, Lauren Stern, Catarina F. Almeida, Barry Slobedman, Allison Abendroth

**Affiliations:** ^1^ Infection, Immunity and Inflammation, School of Medical Sciences, Faculty of Medicine and Health, Charles Perkins Centre, University of Sydney, Sydney, NSW, Australia; ^2^ Sydney Institute for Infectious Diseases , University of Sydney, Sydney, NSW, Australia; ^3^ Department of Microbiology and Immunology, The University of Melbourne, at the Peter Doherty Institute for Infection and Immunity, Melbourne, VIC, Australia

**Keywords:** type I NKT cell, viral infections, CD1d-iNKT cell axis, unconventional innate-like T cell, type I iNKT cell function

## Abstract

Type I NKT cells, also known as Invariant Natural Killer T (iNKT) cells, are a subpopulation of unconventional, innate-like T (ILT) cells which can proficiently influence downstream immune effector functions. Type I NKT cells express a semi-invariant αβ T cell receptor (TCR) that recognises lipid-based ligands specifically presented by the non-classical cluster of differentiation (CD1) protein d (CD1d) molecule. Due to their potent immunomodulatory functional capacity, type I NKT cells are being increasingly considered in prophylactic and therapeutic approaches towards various diseases, including as vaccine-adjuvants. As viruses do not encode lipid synthesis, it is surprising that many studies have shown that some viruses can directly impede type I NKT activation through downregulating CD1d expression. Therefore, in order to harness type I NKT cells for potential anti-viral therapeutic uses, it is critical that we fully appreciate how the CD1d-iNKT cell axis interacts with viral immunity. In this review, we examine clinical findings that underpin the importance of type I NKT cell function in viral infections. This review also explores how certain viruses employ immunoevasive mechanisms and directly encode functions to target CD1d expression and type I NKT cell function. Overall, we suggest that the CD1d-iNKT cell axis may hold greater gravity within viral infections than what was previously appreciated.

## 1 Introduction

The innate immune system mounts a rapid and widespread response to an array of diverse pathogens or danger signals, but is not considered as efficient at forming a memory response for subsequent pathogen exposure. In contrast, the adaptive immune system recognises specific antigenic signatures, albeit with a slower response, but is pivotal for long-term pathogen control. Conventional T cells are mostly thought of as part of the adaptive immune system and mount highly specific, but slow responses. Natural killer T (NKT) cells are a population of unconventional, innate-like T (ILT) cells that can rapidly respond in an innate-like manner, but also in an adaptive-like manner to further enact more antigen-specific responses. Thus, NKT cells are thought to influence and bridge both arms of the immune system.

Unlike conventional αβ T cells that recognise peptide antigens bound by major histocompatibility complex (MHC) molecules, the NKT cell T cell receptor (TCR) exclusively recognises foreign and self-lipid-based antigens presented by the non-classical cluster of differentiation (CD1) protein d (CD1d) molecule. NKT cells are composed of two subpopulations: type I NKT cells, also commonly known as ‘invariant’ NKT (iNKT) cells, which are the best characterised and predominantly explored for their immune-therapeutic potential; and type II NKT cells, which remain less studied and more poorly understood (1). The type I NKT TCR is a semi-invariant αβ TCR comprised of an invariant TCRα chain, Vα24Jα18, which predominantly pairs with Vβ11 TCRs in humans (2, 3). In mice, this TCR comprises of a Vα14Jα18 chain which typically pairs with Vβ2, Vβ7, or Vβ8 chains (4–6). In addition to murine models, swine have also been greatly valued as models for type I NKT cell research as they share a similar type I NKT cell frequency and tissue distribution to that found in humans (7). The TCR of pig type I NKT cells is characterised by a wide range of Vα, Jα, Vβ and Jβ segments, with a large majority of these corresponding to gene sequences recognised in humans (8).

All type I NKT cells share reactivity towards a common lipid antigen termed α-galactosylceramide (α-GalCer), a well-characterised agonist of type I NKT cell responses (9–11). This has enabled the development of CD1d-loaded α-GalCer tetramers, which have facilitated the specific identification of type I NKT cells and type I NKT cell effector function (6, 12, 13). In contrast, type II NKT cells do not express the semi-invariant type I NKT TCR α chain and do not respond to α-GalCer. Instead, type II NKT cells exhibit a greater TCR sequence diversity (14–16) and recognise other lipids and small sulfa-drug-like molecules, such as benzofuran sulfonates, bound by the CD1d molecule (16, 17). Due to a lack of reagents available to universally identify them, much less is known about type II NKT cell immune effector functions and their therapeutic potential remains understudied (15).

Type I NKT cells can be activated upon TCR engagement with a lipid-loaded CD1d molecule, or upon TCR-independent stimulation in response to innate cytokines such as interleukin (IL)-12 and IL-18 (18, 19). Upon activation, type I NKT cells are able to rapidly secrete a plethora of potent cytokines such as interferon (IFN)-γ, tumour necrosis factor (TNF) and IL-4 (20–24). Type I NKT cells can either directly target infected/cancer cells through cytotoxic activity or indirectly control the effector functions of other immune cells, including but not limited to helping B cells form highly specific antibodies (25–29). Due to their capacity to enhance downstream immune functions, type I NKT cells have been increasingly implicated in a variety of viral infections, with their anti-viral potential being a focal point of this review. Current research also harnesses type I NKT cells in multiple clinical settings including anti-cancer treatments, vaccine adjuvants, and cell-based therapies (30, 31).

Although viruses do not typically encode lipid antigens themselves, they are able to modulate the self-lipids expressed from host cells, which can be differentially recognised by certain NKT cell subsets and can affect the cytokine environment upon infection, thus influencing NKT cell responses (type I and type II) (32–34). In agreement, lipodomics studies have shown that viral infections trigger endoplasmic reticulum (ER) stress (35) which can in turn lead to the accumulation of certain CD1d-bound self-lipids (36) that are recognised by the type I NKT TCR (37). This suggests a role for type I NKT cells in viral surveillance through sensing cellular stress (35, 36). Thus, it could be possible that viruses may indirectly modulate CD1d antigen expression of these lipids and inhibit type I NKT cell function to circumvent their anti-viral capacity (38, 39). Considering the profound importance that type I NKT cells may play in viral infections, it is imperative to study the mechanisms through which viruses can either elicit or avoid immune responses through type I NKT cell interaction.

## 2 Type I NKT cells in viral infections

### 2.1 Deficiencies in CD1d molecule expression and Type I NKT cells predispose individuals to exacerbated viral infections

Severe viral infections are more commonly experienced in individuals with weakened and compromised immune capacities (40). Exacerbated symptoms following viral infection have been particularly observed in individuals with decreased CD1d molecule expression or type I NKT cell deficiencies (41–44), underscoring the importance of the CD1d-iNKT cell axis in controlling viral infections.

Varicella zoster virus (VZV) is a highly common alphaherpesvirus with an approximate 90% worldwide seroprevalence (45). In individuals who are latently infected with VZV, periods of diminished VZV-specific immunity results in VZV reactivation (46). VZV reactivation commonly manifests as a painful, unilateral rash (known as herpes zoster/shingles), which typically only occurs once or twice in an immunocompetent individuals’ lifetime (47). Interestingly, individuals who have experienced multiple VZV reactivations exhibited a stark decrease in peripheral type I NKT cell numbers, with residual type I NKT cells skewed to an inhibitory phenotype by higher expression of the inhibitory receptor CD158a (43). IL-2 enhances the functional activity of NK cells and subsequently upregulates CD158a in an attempt to then regulate any cytotoxic repercussions of this activation (48). Interestingly, IL-2 has been readily detectable in varicella patients (49). Thus, the increased inhibitory profile of residual type I NKT cells in zoster patients may be IL-2-dependent and a possible consequence of repeated activation/stimulation. Aimed to increase VZV-specific immunity, primary varicella vaccines, such as Varivax, and booster doses are generally well-tolerated prophylaxis methods (50–52). It is mainly immunodeficient individuals that experience symptoms which are adverse and potentially life-threatening post-vaccination (53, 54). Following vaccination with an attenuated Oka-strain varicella vaccine, two children experienced severe respiratory distress and painful papulovesicular rashes (41, 42). Upon lymphocyte analysis, it was revealed that both patients exhibited a genetic deficiency and dysfunction of type I NKT cell populations, with one patient also deficient in CD1d expression. These clinical findings are consistent with the proposal that the CD1d-iNKT cell axis commands a critical role in VZV resolution.

Mutations in the SH2D1A gene causes defective functioning of the signalling lymphocyte activation molecules (SLAM) -Associated Protein (SAP). SAP is necessary for T and NK cell function and has further been implicated in type I NKT cell development and function (55). Patients with X-linked lymphoproliferative (XLP) 1 disease who have a mutated SH2D1A gene, exhibited a stark absence of type I NKT cells, with no apparent paucity of other lymphocyte populations (55). A child with XLP1 had presented with Epstein-Barr virus (EBV) infection, which then rapidly developed into EBV encephalitis (44). This clinical finding suggests a correlation between the absence and dysfunction of type I NKT cells, and an exacerbated EBV infection.

Recent data has shown that NKT cells also hold great significance in viral control in the context of human transplantation, and thus transplantation success (56). Allografts with a higher abundance of type I NKT cells resulted in a decreased human cytomegalovirus (HCMV) reactivation rate post-allogeneic hematopoietic cell transplantation (HCT), with the association to CD1d expression in these allografts still unknown (56). Accordingly, the secretion of IFN-γ, perforin, and granzyme B from activated iNKT cells had lead to liver damage (57), but had also facilitated cytotoxic T cell activation and thus hepatitis B virus (HBV) inhibition (58). On the other hand, a rat model of hepatitis C virus (HCV) -related virus infection showed that type I NKT cells, which are biased to type 2 immunity, can limit liver injury while preventing infection (59). The role of type I NKT cells in HCV-related virus infection is further explored in Section 2.2 Type I NKT cell activation and function in viral infection. A murine study using NKT knockout mice showed that NKT cell populations and more specifically their IFN-γ production, are necessary for long-term cardiac allograft acceptance (60). In mice who were previously deficient in NKT cells, the adoptive transfer of NKT cells post-transplantation had ameliorated allograft rejection and prolonged cardiac allograft survival (60). Therefore, understanding what factors drive and control the different type I NKT cell subsets could inform how to safely mitigate viral infection severity in a transplant setting, and promote transplantation success.

The importance of CD1d expression and type I NKT cell activity within viral infections has also been supported by studies using murine models. In murine CMV (MCMV) infected mice which were either CD1d or Jα18 deficient, there was a significant suppression of myeloid progenitor cell numbers and proliferative ability (61). Remarkably, the adoptive transfer of type I NKT cells to Jα18 deficient mice, which were then intraperitoneally infected with MCMV, had rescued their myelosuppression profile and improved myeloid progenitor cell cycling status (61). This study suggests that the absence of CD1d molecule expression and type I NKT cell populations leaves myeloid progenitor cells vulnerable to MCMV-induced suppression.

Type I NKT cells have also demonstrated involvement in herpes simplex virus (HSV) type-1 (HSV-1) infection of mice. Following cutaneous inoculation of HSV-1, CD1d knockout mice exhibited an accelerated development of HSV-1 zosteriform skin lesions and a delayed clearance of virus when compared to wild-type mice (62). Jα281 knockout mice, lacking the type I NKT Vα14-Jα281 TCR, also revealed considerably higher viral loads with a diminished capacity to clear virus (62). However, a subsequent study revealed that the Jα281 knockout mice express lower TCR diversity, which can impact the viral-specific T cell repertoire and potentially other unconventional T cells too, such as Mucosal-Associated Invariant T (MAIT) cells (63). Nonetheless, in an alternate murine study, CD1d knockout mice infected with ocular HSV-1 infections displayed exacerbated eye inflammation with a delayed disease clearance (64). As the absence of CD1d molecule expression impedes type I NKT cell development in the thymus of mice (65) and pigs (66), these studies collectively suggest that a lack of CD1d molecule and NKT cell functionality enhances severe viral dissemination. The range of NKT cell functional responses against HSV infection is further explored later in this review.

Overall, many clinical findings and murine studies have established that a deficiency in CD1d molecule expression and type I NKT cell frequencies can leave hosts vulnerable to severe viral dissemination and reactivation. It is thus evident that the CD1d-iNKT cell axis instructs a profound immune response which may be necessary in defending the host from an aggravated viral infection.

### 2.2 Type I NKT cell activation and function in viral infections

The aforementioned studies suggest the involvement and importance of type I NKT cells in anti-viral immune responses. Conversely, multiple studies also report that viral infections modulate the activation and function of type I NKT cells.

#### 2.2.1 Severe acute respiratory syndrome coronavirus 2 (SARS-CoV-2)

SARS-CoV-2 is the virus responsible for COVID-19. A marked depletion of type I NKT cells was observed in the peripheral blood samples of SARS-CoV-2 infected patients, a finding which was found to be independent of CD1d downregulation during infection. This depletion of type I NKT cells was likely a result of SARS-CoV-2 spike protein binding to the type I NKT cell TCR, and causing cellular activation, exhaustion, and apoptosis since existing type I NKT cells expressed higher levels of the exhaustive marker Tim-3 (67). Further studies between convalescent and uninfected patient cohorts revealed that SARS-CoV-2 infected individuals specifically showed a striking reduction in type I NKT cell frequency (68). There was no reduction in conventional T cell frequencies which suggests that type I NKT cells may be more vulnerable to depletion in SARS-CoV-2 infection. In mouse models, the SARS-CoV-2 envelope (E) protein was also found to suppress activation and effector function of type I NKT cells (39). However, when mice were treated with α-GalCer prior to SARS-CoV-2 intranasal infection, they exhibited a decreased viral titre and improved survival rates. Thus, although SARS-CoV-2 can substantially impede type I NKT cell functionality, these findings propose that activated type I NKT cells hold an immunoprotective role against SARS-CoV-2. Interestingly, type I NKT cells expressed a greater activation profile in severe COVID-19 patients (69). A higher CD69 expression level was positively correlated with plasma levels of IL-18, which has been established as a potent activator of type I NKT cells (69, 70). Albeit type I NKT cells from SARS-CoV-2-infected patients did produce less IFN-γ than those from healthy control donors, suggesting that despite a persistent activation profile, type I NKT cells expressed a mitigated functional profile. The substantial activation of type I NKT cells throughout SARS-CoV-2 disease progression is likely to be cytokine-dependent, as well as correlated to spike protein binding, and suggests an intricate balance between activation and functional loss of type I NKT cells in SARS-CoV-2 infection.

#### 2.2.2 Herpes Simplex Virus type -1 (HSV-1)

Epidermal keratinocytes express substantial levels of CD1d and are a primary site of infection by the herpesvirus HSV-1. The co-culture of human type I NKT cells with HSV-1 infected human keratinocytes showed that HSV-1 was able to extensively shut down both the cytokine- and TCR-dependent activation of human type I NKT cells, resulting in an impaired cytokine output (38). However, HSV-1 infected keratinocytes do not exhibit CD1d downregulation which suggests that this weakened functional phenotype of type I NKT cells following contact with HSV-1 infected keratinocytes is independent of CD1d downregulation (38). In contrast to SARS-CoV-2 infection, type I NKT cell function is not rescued by α-GalCer treatment after co-culture with HSV-1 infected keratinocytes (38). Recent studies reveal that in HSV-1 infected human keratinocytes, the IL-15/IL-15 receptor-α (IL-15R-α) complex is rapidly upregulated and then subsequently downregulated with prolonged infection (71). Remarkably, the profound downregulation of the IL-15/IL-15R-α complex by HSV-1 infection was counteracted by IFN-γ production from type I NKT cells. The co-culture of type I NKT cells with HSV-1 infected keratinocytes also resulted in fewer keratinocytes expressing the HSV-1 envelope glycoprotein D (gD) (71). These novel reports represent a new perspective of how type I NKT cells may be able to counteract the modulatory mechanisms of viruses and exert anti-viral activity.

#### 2.2.3 Hepatitis C virus (HCV)

The infection of liver tissue with HCV often results in detrimental inflammation and with no viral clearance, will eventually lead to chronic HCV (72). A patient cohort study indicated that the progression of acute HCV infection to chronic HCV infection is strongly correlated with an elevated activation profile of peripheral blood type I NKT cells as well as pro-inflammatory Type II NKT cells (73), which could possibly be a result of the upregulated CD1d expression present in HCV infection of the liver (74, 135). This suggests that the elevated proinflammatory cytokine milieu of type I NKT cells during HCV infection may contribute to the aggravated liver damage sustained during the progressing stages of HCV infection. However, recent studies have utilised a HCV-related hepacivirus murine model with CD1d knockout mice to explore how the type I NKT cell cytokine response could simultaneously mediate liver pathology and influence hepacivirus-specific CD8^+^ T cells (59). Here, it was deduced that a paucity of liver type I NKT cells led to heightened tissue damage during hepacivirus infection despite previous findings indicating that type I NKT cell function could be associated with liver pathology. These type I NKT cell deficient mice also experienced an exacerbated CD8^+^ T cell response, suggesting that type I NKT cells do offer an immunoprotective role during hepatic viral infection. Furthermore, the production of type 2 cytokines IL-4 and IL-13 from activated type I NKT cell subsets indicated a skewing towards an NKT2 profile (59). These results suggest that type I NKT cells could more specifically serve a regulatory role in viral infections such that the effector functions of hepacivirus-specific T cells, and potentially HCV-specific T cells too, are controlled to limit liver damage.

#### 2.2.4 Influenza A virus (IAV)

IAV is a common respiratory virus that can efficiently infect swine, birds, and humans. From an evolutionary standpoint, the success of IAV within global populations is a result of the virus’ ability to constantly and rapidly produce antigenically distinct viral strains (75). The morbidity and mortality associated with IAV is also related to a substantial viral titre and a destabilising overproduction of cytokines (76). To determine whether type I NKT cells could reduce IAV load and regulate the cytokine production in IAV infection, mice were intraperitoneally administered with α-GalCer concurrent to intranasal IAV inoculation (77). In treated mice, viral titre was significantly lower and body weight also remained more consistent over the course of infection compared to untreated mice. This improved disease outcome was likely a result of the activation and subsequent migration of type I NKT cells from the liver to the lungs, as there was a drop in liver type I NKT cell frequency but a significant increase in blood and lung type I NKT cell frequencies (77). The contribution of activated type I NKT cells to anti-viral immunity is similarly conveyed through IAV-infected CD1d knockout and type I NKT cell deficient mice experiencing an increased IAV titre compared to wild-type mice (78). This is suggested to be correlated to increased myeloid-directed suppressor cell (MDSC) activity, which are a cell-type capable of suppressing T cell functionality and thus cell-mediated anti-IAV immunity. Upon adoptive transfer of type I NKT cells into previously type I NKT cell deficient mice, the suppressive capacity of MDSCs was no longer observed as mice experienced a reduced viral titre, thus indicating the importance of type I NKT cells in controlling MDSC responses in viral environments (78). Type I NKT cells prepared from IAV-infected mice also expressed a higher level of IFN-γ and IL-22 transcripts. This functional output is subsequent to IAV-infected dendritic cells (DCs) activating type I NKT cells via toll-like receptor (TLR)-7/MYD88 signalling and type I NKT cell recognition of secreted IL-1β and IL-23 from IAV-infected DCs (79). The release of IL-22, a critical Th17-related cytokine, from type I NKT cells had protected lung epithelial cells from IAV-mediated cell death whereas the depletion of IL-22 in mice had exacerbated the pathology of airway epithelium (79). In swine models, IAV infection had resulted in an increased frequency and activation of type I NKT cells within blood, lung lymph nodes, and broncho-alveolar lavage (80). It is likely that type I NKT cells may be instrumental in IAV infection of swine as these tissues are all notable in IAV pathology, however further research into the precise role of swine type I NKT cells in anti-IAV immunity is still necessary. These studies thus suggest that type I NKT cells are functionally dynamic in their ability to serve both an anti-viral and protective role against IAV infection.

## 3 Viral immunomodulation of the CD1d antigen presentation pathway

**Figure 1:**
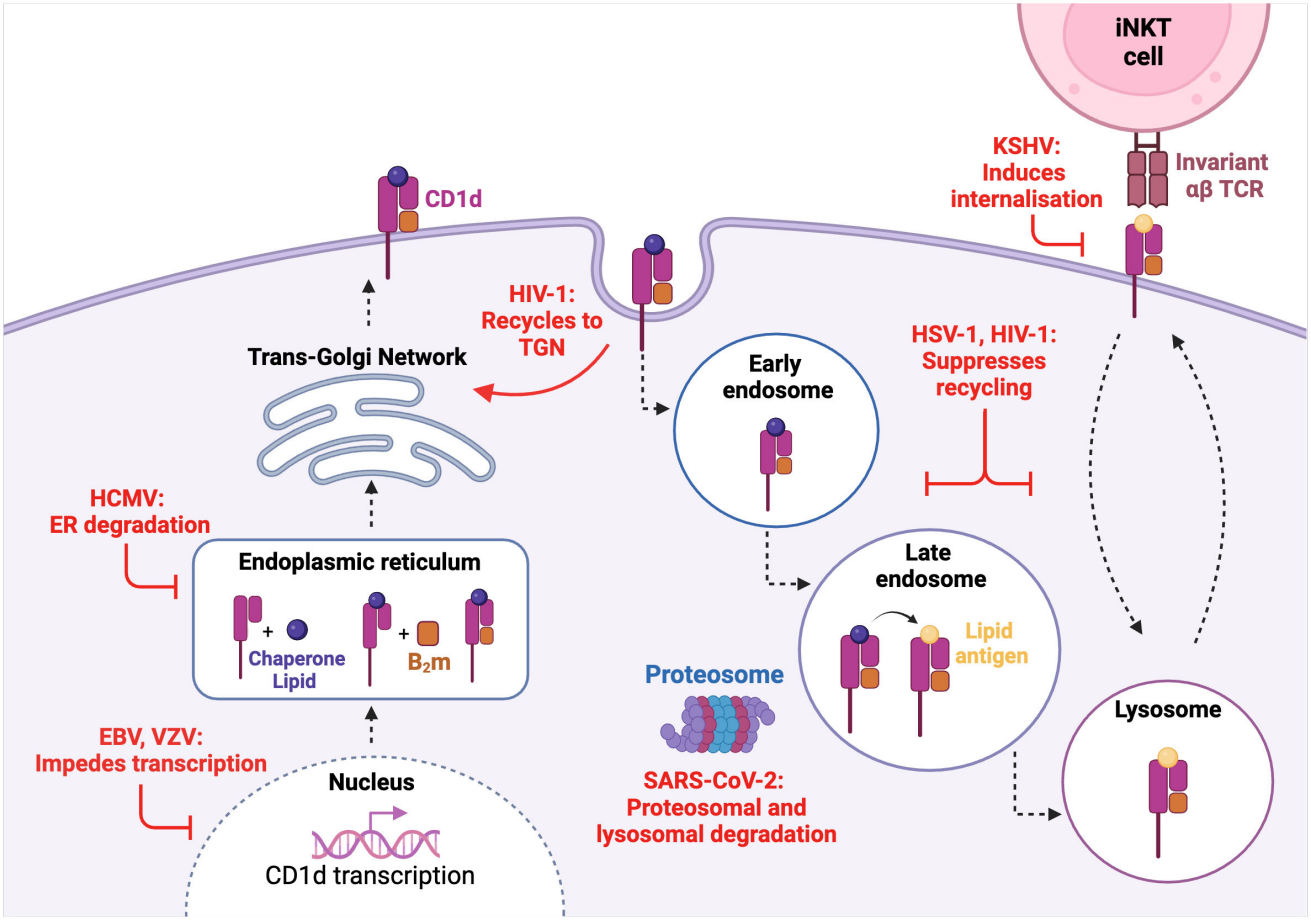
Viral interference of the CD1d molecule biosynthesis and recycling pathway. VZV and EBV impede CD1d transcription while HCMV induces CD1d molecular degradation in the enoplasmic reticulum (ER). HIV-1 and HSV-1 both suppress CD1d cell-surface recycling with HIV-1 also being shown to recycle CD1d back to the trans-golgi network (TGN). SARS-CoV-2 induces both proteasomal and lysosomal degradation of CD1d. KSHV induces CD1d endocytosis and internalisation.

Due to the efficient viral clearance enacted by conventional CD4^+^ and CD8^+^ T cells, it is unsurprising that a myriad of viruses targets the classical antigen presentation pathways of both MHC class -I and -II molecules (81–84). Extensive research has shown that numerous viral infections also impact CD1d antigen presentation and thus type I NKT cell effector function, despite viruses not typically encoding lipid ligands (**Figure 1**).

SARS-CoV-2 is known to be highly successful at immune evasion and suppression. Observed in a human kidney epithelial cell-line, the SARS-CoV-2 envelope (E) protein was found to be responsible for the specific downregulation of mature CD1d molecules (39). As inhibition of proteasomal and lysosomal activity rescued the presence of mature CD1d, it was deduced that the downregulation of CD1d by SARS-CoV-2 is mediated by proteasomal and lysosomal-mediated degradation. However, as aforementioned, previous studies of human peripheral blood from SARS-CoV-2 infected patients showed no CD1d downregulation, thus prompting further research into SARS-CoV-2-mediated modulation of CD1d across different cell-types.

Human immunodeficiency virus (HIV) is an intensively researched virus which mainly infects CD4^+^ T cells, leading to the destruction of cell-mediated immunity and thus impairing the body’s overall immune response. In addition to the finding that CD4^+^ type I NKT cells are also permissive to HIV-1 infection (85), it has also been shown that HIV-1 infection can interfere with CD1d expression and thus, CD1d-dependent activation of type I NKT cells (86, 87). Jurkat cells, which are an immortalised human T cell-line, were infected with GFP-HIV-1, resulting in CD1d being internalised and recycled back to the trans-golgi network (TGN) (86). Interestingly, in GFP-HIV-1 Nef deficient infections, there was minimal CD1d downregulation thus indicating that the immunomodulation of CD1d expression in HIV-1 infection is Nef-dependent. Upon replacing the tyrosine residues in the CD1d molecule cytoplasmic tail with alanine, CD1d expression was not impaired which proposes that the tyrosine-based residues of CD1d are the target of Nef-dependent CD1d internalisation. In HIV-1-infected DCs, the interaction of viral protein U (Vpu) with CD1d resulted in CD1d recycling and retention within the early endosome, thus inhibiting cell-surface presentation (87). Further investigation revealed that Vpu does not alter the rate of CD1d internalisation, but rather prevents the ability of CD1d to be subsequently recycled back to the cell-surface. Patients with HIV-1 are reported to display a reduced abundance of CD4^+^ type I NKT cells (88, 89), which prompts that both the Nef- and Vpu-mediated retention of CD1d could be partially involved in lowered type I NKT cell activation.

The Herpesviruses family is highly ubiquitous and successful, a favourable outcome which is largely underscored by the ability of these viruses to manipulate and evade the host immune response to establish a life-long latent infection (90). Multiple herpesviruses target CD1d expression including HCMV, Kaposi-sarcoma associated herpesvirus (KSHV), EBV, VZV, and HSV-1. In contrast to SARS-CoV-2, the immature form of CD1d is more vulnerable to viral US2-mediated, ubiquitin-dependent proteasomal degradation in HCMV infection (91). The ubiquitination of the CD1d cytoplasmic tail by KSHV induces endocytosis and thus downregulation of cell-surface CD1d (92). Surprisingly, the modulator of immune recognition (MIR) -induced downregulation of CD1d in KSHV infection does not seem to heavily enhance lysosomal degradation, a mechanism that is commonly triggered upon ubiquitin-dependent internalisation (93). This suggests that although CD1d expression is hampered, KSHV has less involvement in its molecular degradation, which seems to be distinct to other viruses studied. During productive infection of human B cells with EBV, a gammaherpesvirus closely related to KSHV, the degradation of CD1d at a transcriptional level by the EBV shutoff protein BFL5 had been reported (94). Recently, VZV has also been shown to downregulate CD1d, which was evident at both a transcript and protein level (95). This downregulated phenotype was observed in both viral antigen-positive cells and VZV-exposed cells that remained viral antigen-negative, a phenomenon unique to VZV infection. This finding is of particular importance given that ‘bystander’ cells are also targeted by VZV in order to inhibit CD1d expression, implying that viral-mediated modulation is not restricted to VZV-infected cells only.

The viral HSV-1 proteins glycoprotein B (gB) and serine-threonine kinase (US3) have also been shown to inhibit the recycling capacity of CD1d in immortalised HeLa cells, thus suppressing type I NKT cell activation (96, 97). As previously discussed, HSV-1 infection of human keratinocytes does not downregulate cell-surface CD1d expression (38). Intriguingly, in human DCs infected with low titres of HSV-1, CD1d expression was upregulated (98). Downregulation of CD1d on HSV-1 infected DCs was only identified in cells with high viral titre (98). These results suggest that the viral-mediated modulation of CD1d is not only cell-type/virus dependent, but also reliant on viral titre.

The downregulation of CD1d by viruses has also been shown by human papilloma virus (HPV) (99), vaccinia virus (100, 101) and vesicular stomatitis virus (100). In contrast, HCV infection caused an upregulation of CD1d expression in chronically infected HCV-infected human liver tissue (74). This finding further suggests that other NKT cell types which hold a stronger CD1d- “self-reactive” profile, such as type II NKT cells, may play a role in anti-viral responses or in influencing type I NKT responses. Overall, it is highlighted that multiple viruses directly encode functions to target CD1d expression at various points in the biosynthesis and recycling pathway, which may contribute to the evolutionary success of certain viruses.

## 4 The interplay between lipid metabolism and viral infections

With such stark modulation of CD1d molecule expression and type I NKT cell function by viruses, the intricate relationship between the CD1d-iNKT cell axis and viral infections is evident. However, there remains postulation as to why viruses may target this unconventional immune cell axis despite its inability to recognise viral proteins. Interestingly, it has been recently reviewed that many viruses manipulate the lipid microenvironment of host cells to enhance the viral lifecycle, and that host lipid mediators may also play a role in the innate immune response to viral infections (102). Through manipulation of host lipid synthesis, it is possible that some viruses may indirectly modulate the presentation of endogenous lipid antigens on CD1d in infected cells, though this remains an understudied area that requires further research.

Hepatic steatosis is a common hallmark of pathology in chronic HCV infection and is characterised by the excess build-up of fat in liver cells. For HCV to efficiently replicate and spread, the HCV Core protein, the tail-interacting protein 47 (TIP47), and the non-structural viral protein 5A (NS5A) all cooperate to transfer viral RNA to lipid droplets (LDs) (103). These LDs act as sites for the construction and assembly of *de novo* virions, in which the Core-dependent recruitment of nonstructural (NS) proteins and replication complexes facilitates HCV production (104). Recent studies have established that although HCV infection does induce LD accumulation in a human hepatic cell line, the increased LD accumulation is not associated with greater levels of HCV Core protein activity (105). Therefore, the accumulation of intracellular LDs in HCV infection is not a direct result of HCV replication, which necessitates further investigation into the mechanisms behind the modulation of lipids during HCV infection. The increased presence of intracellular lipids upon HCV infection may ultimately increase the likelihood of the TCR-dependent activation of type I NKT cells, however, this remains undetermined and warrants further study. Given the potential regulatory role that type I NKT cells may play in HCV pathogenesis (59), we present a valid rationale as to why viruses such as HCV could target a lipid detecting effector cell or a lipid antigen presentation molecule, such as CD1d.

On a similar note, it has been demonstrated that SARS-CoV-2-infected primary human monocytes upregulate lipid metabolism and display an increased accumulation of intracellular LDs, which facilitate viral replication (106). Interestingly, the inhibition of LD synthesis decreases viral progeny production in SARS-CoV-2-infected monocytes and impedes SARS-CoV-2-induced cell death (106). This correlation between SARS-CoV-2-induced lipid accumulation and endogenous lipid ligand availability for type I NKT cell recognition is highly relevant. As mentioned earlier, type I NKT cells are also able to be activated via a cytokine-dependent manner, specifically through IL-12 and IL-18 detection (70). To this end, SARS-CoV-2 infected monocytes exhibit an increased production of IL-12, while the inhibition of lipid synthesis downregulates IL-12 secretion (106). This suggests that the viral replication enabled by lipid synthesis may contribute to the IL-12 production from SARS-CoV-2 infected monocytes. Ultimately, the multi-faceted shut-down of the CD1d-iNKT cell axis by SARS-CoV-2 represents an immune evasion strategy to potentially counteract the increased lipid synthesis needed for viral spread, and thus the potential increase of type I NKT cell surveillance.

While some viruses may manipulate LD production to increase viral replication, it has recently been proposed that LD formation may also hold an anti-viral role too (107). Interestingly, the induction of LD formation following viral infection was exhibited by IAV, HSV-1, Zika virus (ZIKV), and Dengue virus (DENV). IAV infection of human THP-1 monocytes, and HSV-1, ZIKV, and DENV infection of immortalised astrocytes showed that an increased LD accumulation correlated with an enhanced IFN response and thus, a decrease in viral replication (107). Such findings implicate LD accumulation as a possible immune defence mechanism implemented to restrict viral replication rather than to solely facilitate it.

**Figure 2:**
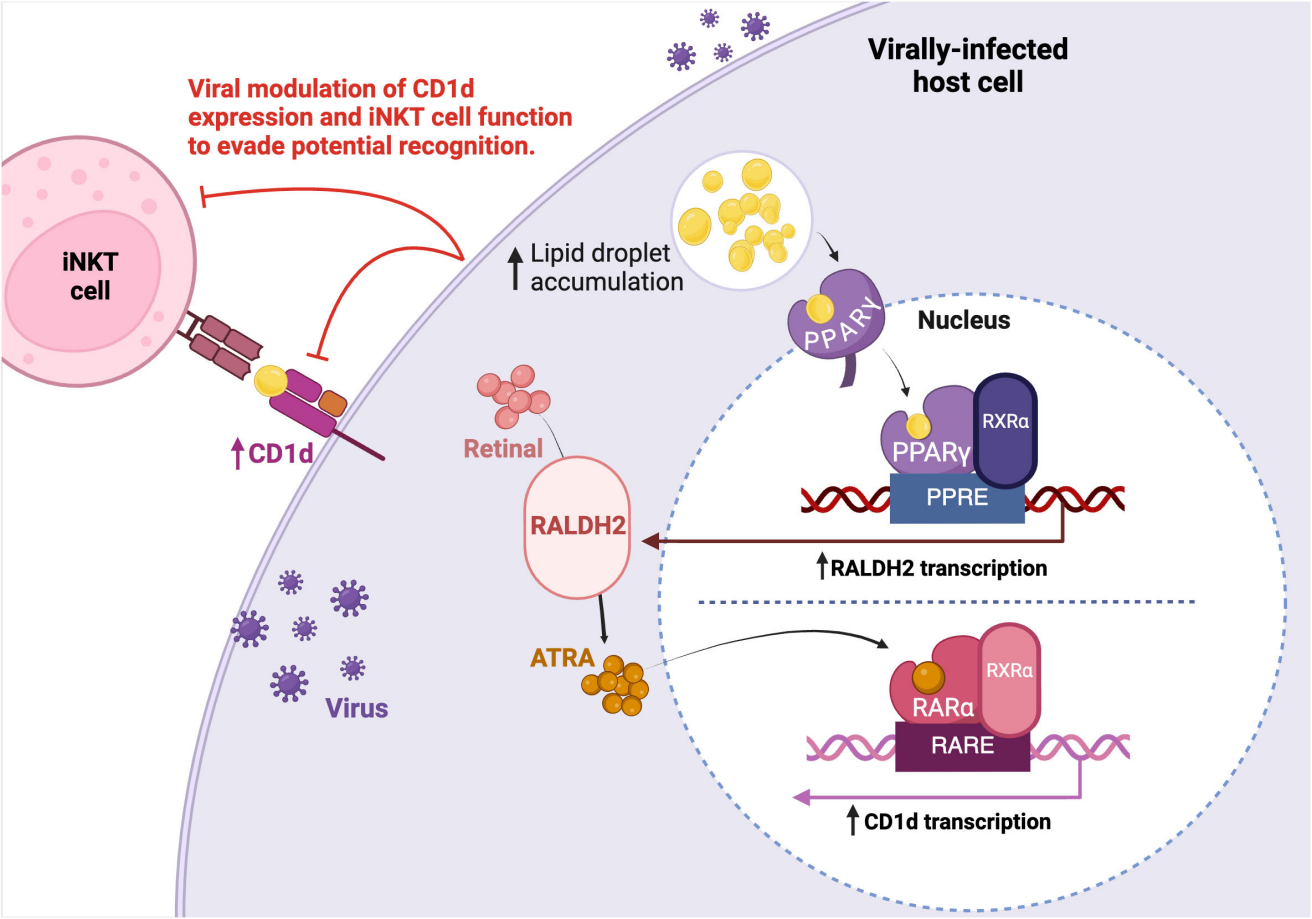
Viral modulation of host cell lipid metabolism. Multiple viruses have been identified to increase lipid droplet (LD) accumulation to facilitate replication. Upon increased LD accumulation, LDs bind to the PPARγ nuclear hormone receptor. PPARγ, which is associated to RXRα, binds to and enhances PPRE promoter activity to upregulate RALDH2 transcription. Increased retinal metabolism through RALDH2 activity leads to an increase of ATRA. ATRA activates the RARα receptor which is bound to the putative RARE promoter sequence found within the CD1d target gene, resulting in greater CD1d transcription. The increased lipid accumulation in consequence of viral replication may indirectly upregulate CD1d expression and thus, the viral modulation of lipid metabolism could promote TCR-dependent type I NKT cell recognition of an infected cell. Therefore, viruses may target CD1d expression and type I NKT cell function to evade potential recognition.

Although certain viruses exploit lipid synthesis to enable viral replication and dissemination, the connection to type I NKT cell activation must still be determined. Upon the detection of lipid ligands such as fatty acids, peroxisome proliferator-activated receptor (PPAR)γ, a lipid-activated transcription factor, is stimulated in DCs (108). PPARγ subsequentially triggers the transcription of retinaldehyde dehydrogenase type 2 (RALDH2) through the promotor activity of peroxisome proliferator responsive element (PPRE). This subsequently increases the abundance of all-trans retinoic acid (ATRA). ATRA then binds and activates the retinoic acid receptor (RAR)α which is found within the CD1d promoter site (109). Therefore, the binding of ATRA to RARα upregulates CD1d transcription and thus, molecular expression (**Figure 2**). As aforementioned, upregulated CD1d expression in a viral environment was indeed reported in HCV infection of human liver tissue (74). While increased lipid metabolism may facilitate *de novo* virion synthesis, the resulting LD accumulation may also indirectly lead to CD1d molecule upregulation and consequently, leave infected cells vulnerable to detection by type I NKT cells. Therefore, the immunomodulation of the CD1d-iNKT cell pathway by certain viruses may stand as an attempt to circumvent the anti-viral capacity of activated type I NKT cells.

## 5 Type I NKT cells in anti-viral immunotherapy and prophylaxis

The use of type I NKT cells in cancer therapeutic approaches is underpinned by their multifaceted ability to activate and enhance anti-tumour immunity. Such therapeutic advances have been seen through allogeneic human stem cell (HSC)-engineered type I NKT cells which have been able to induce potent anti-tumour NK cell activity (110). Moreover, type I NKT cells do not risk graft-versus-host disease (GVHD) following allogenic cancer therapy because they are unresponsive to mismatched MHC molecules between donor and patient, thus proving valuable in anti-tumour therapy applications (111). Such anti-tumour therapeutic advances also include generating chimeric antigen receptor (CAR)-type I NKT cells (30), in which these allogeneic CAR-type I NKT cells selectively target immunosuppressive cells in tumour environments (112), and also the *ex-vivo* expansion and activation of autologous iNKT cells (113). Induced pluripotent stem cell (iPSC)-derived type I NKT cells, which hold a similar genotype and functional profile to primary type I NKT cells, have also shown strong anti-tumour capacity and reduce the limitation of low type I NKT cell frequency in human peripheral blood (114).

Given the importance that type I NKT cells may also play in anti-viral immunity, type I NKT cells are now being harnessed for viral immunotherapy and prophylactic applications. As explored more specifically in this review, the anti-viral therapeutic use of type I NKT cells is being exploited through the adoptive administration of type I NKT cells to mediate viral pathology (115) and through the administration of glycolipid analogues as vaccine adjuvants (115–117).

Allogenic cell-based therapy, where a single donor’s immune cells are modified and introduced back into multiple patients’ blood, is emerging as a promising immunotherapy approach. AgenT-797 is an allogeneic, *ex-vivo* expanded type I NKT cell product (115). Preliminary clinical use against acute respiratory distress syndrome (ARDS) induced by SARS-CoV-2, has shown value in secondary infection prevention and rescue of exhausted T cells (115). Moreso, the key markers of cytokine response syndrome (CRS) were unchanged, with the general cytokine response post-administration favouring an anti-inflammatory profile. Such results indicate that agenT-797 has a dual role in preventing both virus and immune-mediated pathogenesis in SARS-CoV-2 induction of ARDS. The success of cellular therapeutic approaches for viral infections is underpinned by their longevity and persistence within the hosts’ immune system post-administration. In this respect, agenT-797 remained detectable within patient blood and bronchoalveolar lavage (BAL) throughout hospitalisation, with patients who received cardiopulmonary bypass sustaining a stronger retention (115).

IL-4, initially coined as B cell growth factor-1 (BSF-1), plays a significant role in B cell activation and differentiation, and thus is partly responsible for antibody secretion (118, 119). During early stages of influenza infection, type I NKT cells have been found to comprise approximately 70% of the IL-4 producing cells in patient lymph node samples and thus are critical for infection resolution (120). The genetic patterns of type I NKT cells and IL-4 secretion have also corresponded with the abundance of antibodies in macaques infected with ZIKV (120). Recently, a glycolipid agonist adjuvanted to the SARS-CoV-2 RBD-Fc protein ‘αGC-CPOEt’ has shown promise as an effective SARS-CoV-2 vaccine adjuvant in murine models, with the ability to induce a greater secretion of IL-4 from type I NKT cells when compared to a vaccine adjuvanted by α-GalCer (116). Multiple administrations of αGC-CPOEt-adjuvanted vaccinations resulted in increased levels of neutralising antibodies against SARS-CoV-2 (116). This resolves a setback presented by α-GalCer adjuvanted vaccines as multiple exposures to α-GalCer may stun type I NKT cells into anergy and unresponsiveness (121).

A prominent hurdle of vaccine production is the constantly changing SARS-CoV-2 variants which hold distinct antigenic profiles from existing vaccine strains. To address this, a novel type I NKT cell agonist 7DW8-5 has recently shown protection against three antigenically distinct mouse-adapted SARS-CoV-2 strains when administered pre-infection (117). 7DW8-5 is an α-GalCer analogue, which through various biological assays was found to be more potent than α-GalCer at activation of type I NKT cells (122). Analysis of the cytokine profile post-administration of 7DW8-5 showed a skewing towards IFN-γ production from type I NKT, NK, T, and γδ T cells (117). Interestingly, in IFN-γ knockout mice, the anti-viral potential of 7DW8-5 was completely lost, which implies that the potent anti-viral effect of 7DW8-5 is dependent on the induction of an IFN-γ response. In testing whether 7DW8-5 induced anergy in type I NKT cells upon secondary administration, it was established that the repeated administration of 7DW8-5 at both low and high doses did not induce anergy and had maintained protective efficacy (117). Although this agonist still requires extensive clinical testing, *in vitro* testing in human type I NKT cells corroborates with the protective adjuvant activity of 7DW8-5 seen in HIV and malaria murine vaccines (122).

Similar to SARS-CoV-2, swine IAV inflicts a major disease burden in pig populations due to the virus’ ability to evolve and develop drug resistance rapidly. Zoonotic IAV strains can also be transmitted to humans and therefore, swine IAV presents a substantial burden for human populations too (123). The intranasal administration of α-GalCer to H1N1 IAV-infected piglets resulted in complete amelioration of body weight, flu symptoms, and IAV-induced destruction of lung architecture (124). Moreover, α-GalCer-treated piglets had significantly reduced IAV titres compared to untreated piglets (124). This reinforces that activated type I NKT cells could serve as an effective, long-term therapeutic target against swine IAV infection, especially as it may prove difficult for IAV to adapt to the broad functionality of type I NKT cells. Within a prophylactic context, the intramuscular and intranasal administration of α-GalCer to pigs prior to IAV infection did not reduce subsequent viral replication or shedding (125). However, more recent studies have examined the efficacy of α-GalCer treatments for IAV infection in comparison to oseltamivir (126), a widely used anti-viral drug that blocks IAV virion release and spread (127). Here, α-GalCer treatment was ineffective at stimulating an anti-IAV immune response in pigs whereas oseltamivir was able to significantly reduce lung immunopathology and viral spread, suggesting that α-GalCer treatment for swine IAV infection may be highly variable (126). The intranasal co-immunisation of mice with α-GalCer and IAV hemagglutinin glytoprotein had offered substantial protection by inducing a strong mucosal immune response (128). Pigs however represent a more translational animal model than mice, so the therapeutic potential of swine type I NKT cells may be a predictor of type I NKT cell therapy effectiveness in humans with IAV and warrants further investigation.

ABX196 is a variant of α-GalCer with a galactosyl 6-deoxy-6-N-acyl modification and produces a more potent agonistic activity in murine type I NKT cells when compared to the super agonist PBS-57 (129). Preclinical studies in ABX196-treated mice had indicated a large production of IFN-γ from type I NKT cells and NK cells, and did not generate substantial toxicity at any doses (129). Due to the monomorphic nature of the CD1d molecule, ABX196 was also able to be assessed in human subjects as a prophylactic vaccine in combination with HBV surface antigen (HBsAg). In a large portion of patients, an effective anti-HBV antibody response was generated, which is especially noteworthy given the poor immunogenicity of HBsAg. This agonist also established sufficient protective immunity against HBV after only one administration. Activation of liver type I NKT cells by ABX196 did induce cytotoxicity and cellular damage, however future studies can focus on altering the systemic delivery system to overcome this side effect. More recently, a conjugate vaccine platform has incorporated both α-GalCer and HBV viral antigens, such that antigen presenting cells are able to simultaneously activate type I NKT cells and HBV-specific CD8^+^ T cells respectively (130). This co-delivery vaccine design had successfully improved viral clearance in a murine model of chronic HBV however efficacy and safety within human populations is still being evaluated (130). In previous human studies, the treatment of α-GalCer alone produced significant immune activation however this was not enough to efficiently clear HBV, suggesting that the co-delivery of α-GalCer and virus-specific antigens may be a better alternative (131). Unlike the polymorphic MHC molecules, CD1d is a monomorphic molecule and is highly conserved between species and individuals (132), thus representing a likely candidate to explore for future therapeutic gain.

Overall, NKT cells are a functionally dynamic and highly competent cell type that are well-documented in their ability to infiltrate tumour microenvironments and secrete anti-tumour cytokines. In addition, glycolipids are demonstrating significant protection and enhanced efficacy as adjuvants for vaccines against murine models of malaria (133) and as combination treatments with antibiotics against tuberculosis (134). Recent data has introduced a multitude of strategies to harness the multifaceted function of type I NKT cells in anti-viral prophylaxis or treatment, with each approach becoming increasingly valuable.

## 6 Concluding remarks

Type I NKT cells and CD1d antigen presentation molecules represent increasingly relevant players in host responses to viral infections. Although a direct causal relationship between the viral manipulation of host lipid metabolism and type I NKT cell function has yet to be meticulously explored, this review presents a perspective as to why viruses could view the CD1d presentation pathway and type I NKT cells as ideal targets for exploitation. In better understanding the intricate interaction between the CD1d-iNKT cell pathway and viral infections, type I NKT cells could be more prominently placed at the forefront of future viral prophylactic and therapeutic approaches, given their ability to quickly secrete cytokines and aid immune responses. Future exploration into this area may also divulge a better understanding of the unexplored role of type II NKT cells in viral infections and thus, reveal a new appreciation for NKT cells in viral infections.
